# CaMKII T286 autophosphorylation is not propagated at basal Ca ^2+^ levels and is required only for the induction phase of LTP

**DOI:** 10.64898/2026.07.27.741023

**Published:** 2026-07-30

**Authors:** Fan-Yi Chao, Nicole L. Rumian, Adam P. Miller, Steven J. Coultrap, Steve L. Reichow, K. Ulrich Bayer

## Abstract

The Ca ^2+^ /calmodulin-dependent protein kinase II (CaMKII) and its autophosphorylation at T286 (P-T286) mediates long-term potentiation (LTP) of synaptic strength. CaMKII forms stable 12-meric holoenzymes, but exchange of its subunits is proposed to propagate the P-T286 state beyond dephosphorylation or degradation of individual subunits. However, a recent study questioned subunit exchange and instead proposed P-T286 propagation by *trans* -holoenzyme phosphorylation. We show here that both subunit exchange and *trans* -holoenzyme P-T286 can occur (although *cis* -holoenzyme P-T286 is much preferred), but that neither mechanism propagates P-T286 at basal cellular Ca ^2+^ levels (50-100 nM) in absence of further Ca ^2+^ -stimuli. Functionally, we show that after theta-burst stimulation (TBS), P-T286 is required only during the first minutes of LTP induction that prime for subsequent LTP expression, but neither for LTP expression itself nor for LTP maintenance. These results indicate that P-T286 of CaMKII does not self-perpetuate and does not mediate storage but computation of synaptic information.

## Full Text Availability

The license terms selected by the author(s) for this preprint version do not permit archiving in PMC. The full text is available from the preprint server.

